# The clinical impact of chromosomal rearrangements with breakpoints upstream of the *SOX9* gene: two novel de novo balanced translocations associated with acampomelic campomelic dysplasia

**DOI:** 10.1186/1471-2350-14-50

**Published:** 2013-05-07

**Authors:** Ana Carolina S Fonseca, Adriano Bonaldi, Débora R Bertola, Chong A Kim, Paulo A Otto, Angela M Vianna-Morgante

**Affiliations:** 1 Departamento de Genética e Biologia Evolutiva, Instituto de Biociências, Universidade de São Paulo, Rua do Matão, 277, São Paulo, SP, 05508-090, Brazil; 2 Unidade de Genética, Instituto da Criança, HC/FMUSP, São Paulo, SP, Brazil

**Keywords:** Apparently balanced translocation, Acampomelic campomelic dysplasia, *SOX9* regulatory region, Testis-specific *SOX9* enhancer

## Abstract

**Background:**

The association of balanced rearrangements with breakpoints near *SOX9* [SRY (sex determining region Y)-box 9] with skeletal abnormalities has been ascribed to the presumptive altering of *SOX9* expression by the direct disruption of regulatory elements, their separation from *SOX9* or the effect of juxtaposed sequences.

**Case presentation:**

We report on two sporadic apparently balanced translocations, t(7;17)(p13;q24) and t(17;20)(q24.3;q11.2), whose carriers have skeletal abnormalities that led to the diagnosis of acampomelic campomelic dysplasia (ACD; MIM 114290). No pathogenic chromosomal imbalances were detected by a-CGH. The chromosome 17 breakpoints were mapped, respectively, 917–855 kb and 601–585 kb upstream of the *SOX9* gene. A distal cluster of balanced rearrangements breakpoints on chromosome 17 associated with *SOX9*-related skeletal disorders has been mapped to a segment 932–789 kb upstream of *SOX9*. In this cluster, the breakpoint of the herein described t(17;20) is the most telomeric to *SOX9*, thus allowing the redefining of the telomeric boundary of the distal breakpoint cluster region related to skeletal disorders to 601–585 kb upstream of *SOX9*. Although both patients have skeletal abnormalities, the t(7;17) carrier presents with relatively mild clinical features, whereas the t(17;20) was detected in a boy with severe broncheomalacia, depending on mechanical ventilation. Balanced and unbalanced rearrangements associated with disorders of sex determination led to the mapping of a regulatory region of *SOX9* function on testicular differentiation to a 517–595 kb interval upstream of *SOX9*, in addition to TESCO (Testis-specific enhancer of *SOX9* core). As the carrier of t(17;20) has an XY sex-chromosome constitution and normal male development for his age, the segment of chromosome 17 distal to the translocation breakpoint should contain the regulatory elements for normal testis development.

**Conclusions:**

These two novel translocations illustrate the clinical variability in carriers of balanced translocations with breakpoints near *SOX9.* The translocation t(17;20) breakpoint provides further evidence for an additional testis-specific *SOX9* enhancer 517 to 595 kb upstream of the *SOX9* gene.

## Background

The *SOX9* [SRY (sex determining region Y)-box 9] gene, mapped at 17q24.3, encodes a transcription factor with a role in chondrogenesis [1], and also required for cellular differentiation in heart, glial cells, neural crest progenitors, notochord, pancreas, inner ear and testis [2]. Mutations in the coding region of *SOX9* cause campomelic dysplasia (CD) [3]. CD is a rare and often lethal disease, characterized by skeletal abnormalities, including shortening and bowing of the long bones, hypoplasia of the scapular and pelvic bones, poor mineralization of thoracic pedicles, 11 pairs of ribs, and narrow iliac bones [4]. Respiratory distress, caused by small thoracic cage and narrow airways from defective tracheobronchial cartilages, is the main cause of death, which occurs mostly in the neonatal period. About 75% of CD patients with a 46,XY karyotype present disorders of sex development with ambiguous or female external genitalia (46,XY DSD) [4]. The abnormal curvature of long bones, the main characteristic of CD, is absent in 10% of patients, and the disease is then called acampomelic campomelic dysplasia (ACD) [5]. Pierre Robin Sequence (PRS) characterized by micrognathia, retroglossia, glossoptosis and posterior cleft palate, is also a feature of CD and ACD [6]. Although the majority of patients carry mutations in the coding region of *SOX9*, balanced rearrangements and deletions with breakpoints mapped upstream of *SOX9* have been associated with CD, ACD and PRS [7-12]. 46,XY DSD has also been described in CD and ACD patients who carry microdeletions and balanced rearrangements upstream of *SOX9*[8,9,11,12]. Isolated 46,XY DSD were also associated with microdeletions upstream of *SOX9*[13,14]. Furthermore, duplications and triplications of segments upstream of *SOX9* have been associated with isolated XX female-to-male disorder of sex development (46,XX DSD) [13,15,16], and an apparently balanced translocation with a breakpoint upstream of *SOX9* was detected in an XX male with minor skeletal defects [17]. More distally mapped duplications upstream of *SOX9* result in brachydactyly-anonychia [18], a defect that is not part of the ACD-CD phenotype. The diversity of skeletal abnormalities and DSD associated with these balanced and unbalanced rearrangements, extending over the 1Mb region upstream of *SOX9*, evidences the complexity of the gene regulatory region [19]. Adding to this, a balanced translocation in an ACD patient [20] and a deletion in a PRS patients [10], both mapped 3′ to *SOX9,* pointed to regulatory elements located downstream of the gene.

Here we report on two ACD patients who carry apparently balanced translocations, t(7;17)(p13;q24) and t(17;20)(q24.3; q11.2). These two novel translocations further illustrate the variability in the severity of phenotypes associated with breakpoints clustered upstream of *SOX9*. Evidence is provided for the regulatory region critical for gonadal *SOX9* expression proposed by Benko et al. [13].

## Case presentation

This study was approved by the Ethic Committee for research involving human subjects at the Biosciences Institute, University of São Paulo.

### Patients

#### Patient 1

The girl, the first child of healthy non consanguineous parents, was delivered at term after cesarean section due to breech presentation. In the first six years of age, she had several episodes of acute bronchitis. Her growth chart showed her stature to be at the 50th centile until the age of 6 years when it gradually started to fall off, and it was around the 25th centile, at the age of 9 years. Neuropsychomotor development was normal. At 12 years of age, she was referred to the Genetic Counseling Service at the University of São Paulo, because of short stature (136.5 cm, 3rd percentile), delayed bone age and skeletal abnormalities that included hypoplastic scapulae, thoracolumbar scoliosis, 11pairs of ribs with hypoplasia of the first four pairs. She was prepubertal. Intellectual development was normal. The chromosome analysis after G-banding revealed an apparently balanced reciprocal translocation between the short arm of chromosome 7 and the long arm of chromosome 17 - t(7;17)(p13;q24). Her parents had normal karyotypes. At 31 years of age, she returned for genetic counseling worried about her risks of affected offspring; her height (159 cm) and weight (54 kg) were around the 25th centile.

#### Patient 2

The boy, the second child of healthy non consanguineous parents, was delivered at term after an uneventful pregnancy. Birth weight was 3350 g (50th centile), length 47 cm (10th centile), and occipitofrontal circumference, 36 cm (50th centile); Apgar scores were 7, 8 and 10. The presence of posterior cleft palate and micro/retrognathia led to the diagnostic hypothesis of Pierre-Robin sequence. Scoliosis was documented by thoracic X-ray. The boy evolved with respiratory distress, requiring oxygen supplementation. At 50 days of age, he was discharged from the hospital, but two weeks later was readmitted with respiratory distress, pallor, and poor general conditions, evolving with apnea and severe bradychardia that resolved with oxygen supplementation and chest compression. He was admitted at the ICU requiring mechanical ventilation. Difficulty in weaning from this support, led to the need of tracheostomy and gastrostomy at 6 months of age; complementary exams disclosed tracheobronchomalacia. Since then, the patient has been under long-term ventilation. He evolved with delayed milestones, sitting up without support at 18 months of age, and walking at the age of 30 months. At 3 years of age, physical examination by one of us (DB) showed weight of 14 kg (25-50th centile), height of 91 cm (10-25th centile) and occipitofrontal circumference of 51 cm (50-98th centile); he had a flat facies, bilateral epicanthus, low nasal bridge with anteverted nares, posterior cleft palate, mild micrognathia; short neck; hypoplastic nipples, and severe kyphoscoliosis. He had normal male genitalia, and proportionate limbs. Speech could not be evaluated, but he showed good social interaction and understanding of orders. Gradual amelioration of his respiratory symptoms was recorded, but he still needed some degree of ventilatory support. Skeletal X-rays documented severe thoracic kyphoscoliosis, and non-mineralized thoracic vertebral pedicles; narrow iliac wings, delayed ischiopubic ossification, femur with flat capital epiphysis, elongated neck, and absent lesser trochanter; short fibula. Chromosome analysis after G-banding revealed an apparently balanced reciprocal translocation between the long arm of chromosome 17 and the long arm of chromosome 20 -t(17;20)(q24.3;q11.2). His parents had normal karyotypes.

### Sequencing of the *SOX9* gene

As *SOX9* point mutations are known to cause ACD, direct sequencing of *SOX9* coding region and intron/exon boundaries were performed in both patients. Sequence reactions were performed with the BigDye Terminator v3.1 Cycle Sequencing Ready Reaction kit (Applied Biosystems, California, USA), and analyzed on an ABI-3730 (Applied Biosystems). No pathogenic mutations were found.

### Mapping of the translocation breakpoints

Fluorescent in situ hybridization (FISH) was performed on metaphases from cultured peripheral blood lymphocytes with BACs selected on the University of California – Santa Cruz – Genome Browser (UCSC; http://genome.ucsc.edu; hg 19; Additional file 1: Table S1 and Additional file 2: Table S2). Probes were labeled with biotin-14-dATP or digoxigenin-11-dUTP by nick translation (Bio/Dig-Nick Translation kit; Roche Diagnostics GmbH, Mannheim, Germany), and were visualized with FITC- or rhodamine-conjugated antibodies, chromosomes being counterstained with 4,6-diamidine-2-phenylindole (DAPI) in VectaShield Mounting Medium (Vector Laboratories, Burlingame, CA, USA). Chromosomal micro imbalances were investigated by array-comparative genomic hybridization (a-CGH) using the Agilent Human Genome 105A CGH Microarray (Agilent Technologies Inc., Santa Clara, CA, USA). The microarray chip was scanned on an Agilent Microarray Scanner. The data were processed by Agilent Feature Extraction software 9.5 and analyzed with Agilent CGH Analytics 3.4 Software with the statistical algorithm ADM-2, and sensitivity threshold 6.7. At least three consecutive oligonucleotides had to have aberrant log2 ratios to be called by the software.

#### Patient 1 – t(7;17)

**Figure 1:**
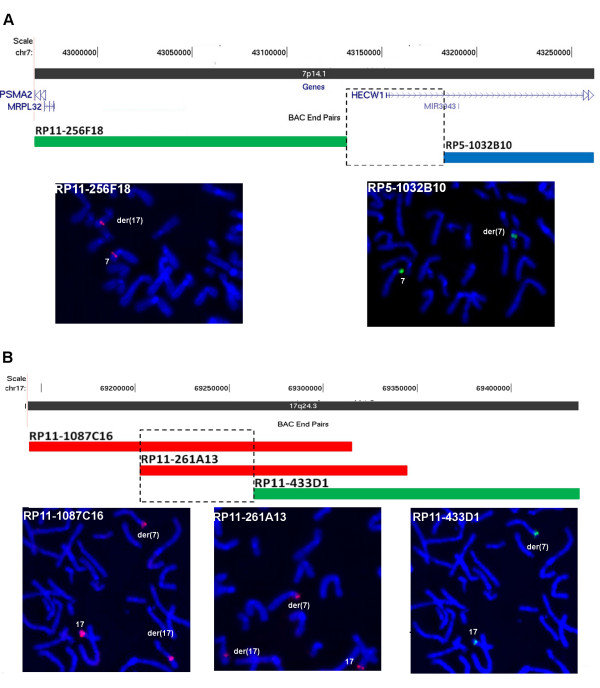
**Analysis of the t(7;17) breakpoint regions on chromosomes 7 and 17.** (**A**) Chromosome 7 clone RP11-256F18 yielded FISH signals on the normal 7 and on the der(17). Clone RP5-1032B10 produced FISH signals on the normal 7 and on the der(7). The 53 kb segment (chr7:43,130,626-43,183,638) between these clones contains the chromosome 7 breakpoint. The *HECW1* gene, partially mapped to this interval, is depicted. (**B**) Chromosome 17 clones RP11-1087C16 and RP11-261A13 produced FISH signals on the normal 17 and on both the der(7) and the der(17); the weaker signal of RP11-1087C16 on the der(7) suggests that the breakpoint occurred at the distal end of this clone. Clone RP11-433D1 yielded FISH signals on the normal 17 and on the der(7). The 62 kb segment of overlap between clones RP11-1087C16 and RP11-261A1, delimited by the sequence of clone RP11-1087C16 that does not overlap with clone RP11-433D1, contains the translocation breakpoint (chr17: 69,201,539-69,262,086). (Based on UCSC Genome Bioinformatics, hg19).

On chromosome 7, the breakpoint was mapped within a ~53 kb interval (chr7:43,130,626-43,183,638; Human GRCh37 assembly, hg19) (Figure 1A and Additional file 1: Table S1). The gene *HECW1* (HECT, C2 and WW domain containing E3 ubiquitin protein ligase 1), which is expressed especially in brain and skeletal muscle, is partially mapped to this interval and might have been interrupted by the breakpoint. On chromosome 17, the breakpoint was mapped to a ~62 kb segment (chr17: 69,201,539-69,262,086), a gene desert region (Figure 1B and Additional file 1: Table S1). No potentially pathogenic chromosomal imbalances were detected by a-CGH.

#### Patient 2 – t(17;20)

**Figure 2:**
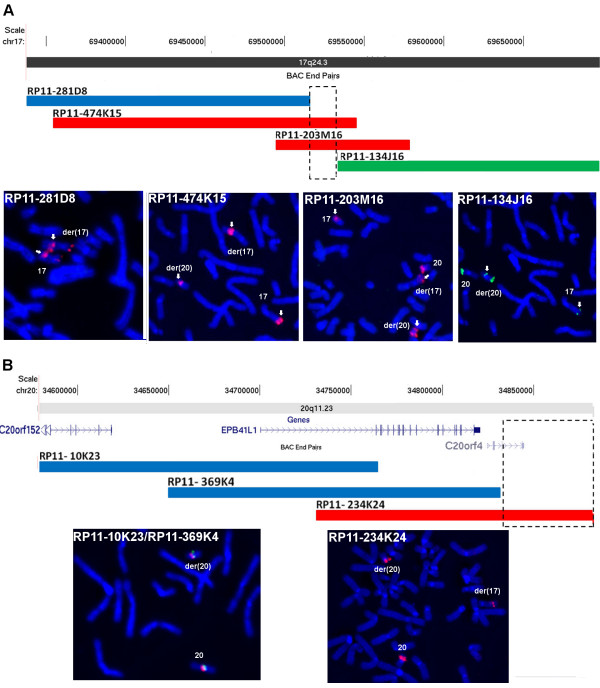
**Analysis of the t(17;20) breakpoint regions on the 17 and 20 chromosomes.** (**A**) Chromosome 17 clone RP11-281D8 yielded FISH signals on the normal chromosome 17 and on the der(17) (arrows).Clones RP11-474K15 and RP11-203M16 produced FISH signals on the normal chromosome 17 and on both derivative chromosomes, der(17) and der(20) (arrows); the stronger signal of RP11-474K15 on the der(17) suggests that the breakpoint occurred on the distal end of this clone. Clone RP11-134J16 yielded FISH signals on the normal chromosome 17 and on the der(20) (arrows). Cross hybridization signals were produced by clones RP11-281D8 (on the short arms of chromosomes 17), RP11-203M16 and RP11-134J16 [on the short arms of chromosomes 20 and der(20)], and RP11-281D8 [on the short arms of chromosomes 17 and der(17)]. The 15 kb segment, overlapped by clones RP11-474K15 and RP11-203M16 and delimited by the sequence between clones RP11-281D8 and RP11-134J16, contains the chromosome 17 breakpoint (chr17: 69,516,104-69,531,685). (**B**) Chromosome 20 clones RP11-10K23 (red) and RP11-369K4 (green) produced FISH signals on the normal chromosome 20 and on the der(20). Clone RP11-234K24 produced FISH signals on the normal chromosome 20 and on both derivative chromosomes, der(17) and der(20); the stronger signal of clone RP11-234K24 on the der(20) suggests that the breakpoint occurred on the distal end of the clone. The 50 kb segment of clone RP11-234K24, delimited by the sequence of clone RP11-234K24 that does not overlap with clones RP11-10K23 and RP11-369K4, contains the translocation breakpoint (chr20: 34,832,526-34,883,260). (Based on UCSC Genome Bioinformatics, hg19).

On chromosome 17 the breakpoint was mapped to a ~16 kb segment (chr17: 69,516,104-69,531,685), a gene desert region (Figure 2A and Additional file 2: Table S2). On chromosome 20, the breakpoint was mapped within a ~50 kb interval (chr20: 34,832,526-34,883,260) (Figure 2B and Additional file 2: Table S2). The C20orf4 (chromosome 20 open reading frame 4) is mapped to this interval and might have been interrupted by the breakpoint. No potentially pathogenic chromosomal imbalances were detected by a-CGH.

## Discussion

**Figure 3:**
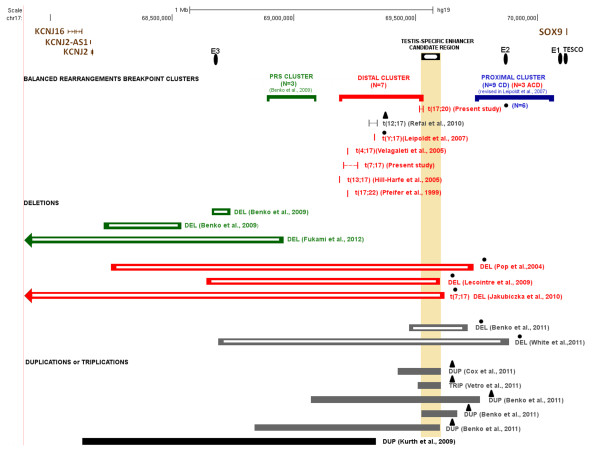
**Chromosomal rearrangements upstream of *SOX9 *presumptively associated with *SOX9 *misregulation.** Balanced and unbalanced rearrangements with breakpoints mapped upstream of *SOX9*, detected in patients with campomelic dysplasia (CD; blue), acampomelic campomelic dysplasia (ACD; red), Pierre Robin Sequence (PRS; green), minor or no skeletal defects (gray) and brachydactyly-anonychia (black) are shown. The centromeric limit of the two deletions represented by arrows extends beyond the region shown. *SOX9* and other nearby genes are depicted. The breakpoints of balanced rearrangements are grouped into proximal, distal and PRS breakpoint clusters, according to their distance from *SOX9*; the herein described t(17;20) redefines the telomeric boundary of the distal cluster (see Table 1 for details). Disorders of sex development (DSD) are indicated: XY SDS (black circle symbol) and XX SDS (black triangle symbol). *SOX9* regulatory elements **E1** and **E2**[21], **TESCO**[22], and **E3**[10] are shown in the upper part of the figure (*black oval symbol*). The candidate region for the **TESTIS-SPECIFIC ENHANCER**[13], represented by the vertical bar, is spanned by all deletions and duplications detected in patients with DSD. (Based on UCSC Genome Bioinformatics, hg19).

Here we describe two carriers of apparently balanced translocations with breakpoints at 17q24.3. Both patients had skeletal defects that led to the diagnosis of acampomelic campomelic dysplasia (ACD). The breakpoints of the t(7:17)(p13;q24) and t(17;20)(q24.3;q11.2) translocations were mapped on chromosome 17, respectively, 855-917 kb and 585–601 kb upstream of *SOX9*. These translocations caused the clinical phenotypes by presumptively altering *SOX9* expression, as other previously described balanced rearrangements upstream of *SOX9* associated with skeletal defects (Figure 3 and Table 1). Based on the clinical presentation of carriers and the distance of breakpoints from the *SOX9* coding sequence, these balanced rearrangements were grouped in three clusters - “proximal”, “distal” and “PRS” [9,10,19]. Breakpoints of balanced rearrangements mapped 50–375 kb and 789–932 kb upstream of the *SOX9* coding sequence, were grouped, respectively, into the proximal and distal clusters [9]. These balanced rearrangements were detected in CD and ACD patients. In addition, a third clustering of balanced rearrangements, associated with the Pierre Robin Sequence (PRS), mapped 1.03-1.26Mb upstream of *SOX9*, [10]. Accordingly, the translocations here described should be included in the distal cluster mapped 789–932 kb upstream of *SOX9*. The translocation t(17;20) breakpoint, however, is more proximal to *SOX9*, thus allowing the redefining of the telomeric boundary of the distal cluster of breakpoints to 585–601 kb upstream of *SOX9*.

**Table 1:** Chromosome 17 balanced rearrangements and deletions in patients with campomelic dysplasia (CD), acampomelic campomelic dysplasia (ACD) and Pierre-Robin sequence (PRS)

**Rearrangements**	**Inheritance**	**Breakpoint distance from *SOX9 *(kb)**		**Breakpoint cluster**	**Phenotype**	**Sex**	**Survival time**	**Reference**
		**5′**	**3′**					
t(7;17)(q34;q25.1)	Sporadic	50		Proximal	CD (Severe campomelia)	F*	>6 years	[23,24]
t(12;17)(q21.32;q24.3-q25.1)	Sporadic	74–88		Proximal	ACD	F*	11 months (diseased)	[25,26]
t(2;17)(q35;q23-q24)	Sporadic	88		Proximal	CD (Severe campomelia)	F*	Abortion	[3,27]
t(9;17)	Sporadic	110–140		Proximal	CD (Severe campomelia)	M	>3 years	[28]
t(13;17)(q22;q25.1)	Sporadic	134–142		Proximal	ACD	F*	>30 years	[7,23,29]
t(1;17)(q42.13;q24.3-q25.1)	Sporadic	173–179		Proximal	CD (Severe campomelia)	F	>6 years	[7,23,29]
t(5;17)(q23.2;q24)	Sporadic	161		Proximal	ACD	?	?	[30]
t(6;17)(q14;q24)	Sporadic	212–224		Proximal	CD (Mild campomelia)	F*	>3 years	[7,29]
t(10;17)(q24;q23)	Sporadic	228–229		Proximal	CD (Severe campomelia)	(M)	>1 year	[7]
t(5;17)(q13.3;q24.2)	Sporadic	288–319		Proximal	CD (Severe campomelia)	F	>12 years	[7]
inv(17)(q11.2;q24.3-q25.1)	Sporadic	70–350		Proximal	CD (Mild campomelia)	F*	>2 years	[4,28]
t(1;17) (q42.1;q24.3)	Sporadic	375		Proximal	CD (Mild campomelia)	(M)	>5 years	[9]
del(17)(q24.3)	Sporadic	380-1.869			ACD	F*	>5 years	[8]
del(17)(q24.3)	Familial	517-1.477			ACD	F*	> 3 and 32 years	[11]
t(7;17)(q33;q24)del(17)(q24.2q24.3)	Sporadic	500-4700			ACD	F*	?	[12]
t(17;20)(q24.3;q11.2)	Sporadic	585-601		Distal	ACD	M	>3 years	Present study
t(12;17)(q14.3;q24.3)	Sporadic	776-811		Distal	Mandibular and malar hypoplasia	M*	> Newborn	[17]
t(Y;17)(q11.2;q24.3)	Sporadic	789		Distal	ACD	F*	>3 years	[9]
t(7;17)(p.13;q24)	Sporadic	855-917		Distal	ACD	F	>32 years	Present study
t(4;17)(q28.3;q24.3)	Familial	899		Distal	ACD	F /M	> 6 and 32 years	[20]
t(13;17)(q22.1;q22.3)	Familial	932		Distal	ACD	F/ M	?	[31]
t(17;22)(q25.1;p11.2)	Sporadic	900		Distal	ACD	{M}	6 years (diseased)	[7,31]
t(5;17)(q15;q24)	Familial	1030-1100		PRS	PRS	F/M	?	[10]
t(2;17)(q32;q24)	Familial	1160-1200		PRS	PRS	F/M	?	[10]
inv(17)(q21.31q24.3)del(17)(q24.3q23)	Sporadic	1160-1460			PRS	M	>1 year	[32]
t(2;17)(q24.1;q24.3)	Familial	1230		PRS	PRS	F /M	?	[10]
del(17)(q24.3)	Familial	1380-1450			PRS	F /M	?	[10]
del(17)(q24.3)	Sporadic	1580-1890			PRS	?	?	[10]
t(4;7;8;17)	Sporadic		1300		ACD	F*	3 weeks (diseased)	[20]
del(17)(q25.1)	Sporadic		1520-1560		PRS	?	?	[10]

*CD* Campomelic Dysplasia, *ACD* Acampomelic Campomelic Dysplasia, *PRS* Pierre Robin Sequence, *DSD* Disorder of Sex Development.

*M* Male, (*M*) Male with hypospadias {M}, Male with small penis M*, 46, XX DSD, *F* Female XX, F*, 46,XY DSD.

The spectrum of phenotypes associated with *SOX9* presumptive misregulation provides insights into the gene regulatory region. As opposed to carriers of *SOX9* point mutations, who usually die in the neonatal period [4], carriers of balanced rearrangements tend to have longer life expectancy (Table 1), an indication that balanced rearrangements that presumptively modify *SOX9* expression generally do not impact *SOX9* expression to the same extent as the inactivation of one *SOX9* allele by point mutations. However, balanced rearrangements with breakpoints in the proximal and distal clusters are associated with CD and ACD, thus indicating that breakpoints mapped 50 kb to 932 kb upstream of *SOX9* modify its expression in craniofacial structures, scapulae, ribs, vertebrae and limbs. The disruption of regulatory elements by breakpoints or their separation from *SOX9* might have altered the gene spatial/temporal expression in carriers of these balanced rearrangements. In fact, the segment 350 kb upstream of the gene contains several *SOX9* putative regulatory elements [21,28] (Figure 3). Nine out of the 12 balanced rearrangements in the proximal cluster were detected in CD patients with mild (three cases) or severe (six cases) bowing of long bones (Table 1). On the other hand, the seven balanced translocations in the distal cluster, including the two novel translocations we describe here, were detected in ACD patients; the exception was a translocation t(12;17) in a patient whose osseous anomalies consisted only of mandibular and malar hypoplasia, and a dysplastic 12th pair of ribs [17] (Table 1). The translocations in this distal cluster would have impacted *SOX9* more distally located regulatory elements, the more proximal ones remaining in cis to *SOX9*, thus resulting in straight long bones. The PRS cluster points to regulatory elements located more than 1Mb upstream of *SOX9* that would control its expression in craniofacial structures; alternatively, these could also be general enhancers affecting only craniofacial structures due to its sensitivity to *SOX9* levels [19].The three reported translocations in the PRS cluster would have affected only the *SOX9* more distally located regulatory elements, and the rest of the gene regulatory region would have remained intact.

Large deletions upstream of *SOX9*, which remove the PRS and distal clusters, were detected in patients with a mild ACD phenotype [8,11,12] similar to that manifested by carriers of balanced translocations with the most distally located breakpoints in the distal cluster– t(13;17) [31], t(4;17) [20], and t(7;17) here described (Table 1 and Figure 3). These rearrangements would have affected the same regulatory element(s). Deletions mapped more than 1Mb upstream of *SOX9* result in PRS [10,32]. Likewise, these deletions would have impacted the same regulatory element(s) as balanced translocations in the PRS cluster.

In addition to isolating the *SOX9* from cis regulatory elements or disrupting these elements, the chromosomal rearrangements might impact *SOX9* expression through a position effect that would explain the phenotype variability of carriers of rearrangements with breakpoints mapped to the same cluster. For instance, the carriers of three balanced translocations in the distal cluster had severe forms of ACD (Figure 3). A patient whose translocation breakpoint mapped 900 kb upstream of *SOX9* died at the age of 6 years consequent to tracheobronchomalacia [7]; the carrier of a translocation with a breakpoint 789 kb from *SOX9* also suffered from severe respiratory distress in early infancy [9], and the herein described carrier of the t(17;20) with a breakpoint 585–601 kb from *SOX9* had severe tracheobroncheomalacia and remained hospitalized on the dependence of mechanical ventilation for proper maintenance of oxygen saturation. The chromosome 17 breakpoint of the two previously published translocations were mapped to close proximity of those of balanced translocations in patients with milder phenotypes, including familial cases (Figure 3; Table 1). The authors proposed that the translocation of *SOX9* gene to heterochromatic regions or its proximity would have resulted in reduction of expression/silencing of the gene. Similarly, the severe phenotype of the carrier of the t(17;20) here described might have resulted from the effect of chromosome 20 sequences on *SOX9* expression.

*SOX9* has a role in sex determination. A *SOX9* testis-specific enhancer, TESCO (Testis-specific Enhancer of *SOX9* Core), was mapped 10–15 kb upstream of *SOX9*[22] (Figure 3). However, deletions upstream of *SOX9* in patients with 46,XY DSD [8,11-14], as well as duplications upstream of *SOX9* in patients with 46,XX DSD [13,15,16,33] do not include TESCO (Figure 3), thus pointing to a second testis-specific enhancer located 517–595 kb upstream of *SOX9*[13]. Loss of this region in deletion carriers resulted in 46,XY DSD, despite the presence of an intact *SRY* gene [8,11-14]. The high expression of *SOX9* would lead to the testicular differentiation in XX males carrying duplication of this region [13,15,16,33]. As the carrier of t(17;20) here described has an XY sex-chromosome constitution and normal male development for his age, the segment of chromosome 17 distal to the translocation breakpoint should contain the regulatory elements necessary for normal testis development, and was translocated to chromosome 20 together with *SOX9*. Thus this translocation breakpoint provides further evidence of a cis-acting testis-specific enhancer located 517 to 585–595 kb upstream of *SOX9*. The isolating of this testis-specific potential enhancer from *SOX9* would explain why the great majority of balanced rearrangements in the proximal cluster, which do not affect TESCO, result in XY DSD or XY males with hypospadia (Table 1; Figure 3). However, two balanced translocations with breakpoints in the distal cluster - the t(Y; 17), carried by an XY female [9] and the t(12;17), carried by an XX male [17], are mapped centromeric to this putative testis specific enhancer (Figure 3). In these two cases, a position effect of the Y heterochromatic block and of chromosome 12 sequences, respectively, would have reduced or increased *SOX9* expression, thus leading to female and male phenotypes, respectively. The low and high level of *SOX9* expression, respectively, would also explain the severe and mild skeletal abnormalities in these two patients [9,17].

## Conclusions

The novel translocations t(7;17) and t(17;20) herein described illustrate the clinical variability in carriers of balanced translocations with breakpoints near *SOX9.* Taken together, the rearrangements affecting segments in the vicinity of *SOX9* point to a regulatory region that control skeletal development possibly extending beyond the 1Mb region upstream of *SOX9*, and to a regulatory region of sex determination, in addition to TESCO, located 517–595 kb upstream of *SOX9*. The breakpoint of the herein described t(17;20) allowed to redefine the telomeric boundary of the distal breakpoint of the cluster region related to skeletal defects to 601–585 kb upstream of *SOX9*. The translocation t(17;20) breakpoint also provides further evidence for an additional testis-specific *SOX9* enhancer 517 to 595 kb upstream of the *SOX9* gene.

## Consent

Written informed consent was obtained from the patient or parents for publication of this clinical data and any accompanying images.

## Abbreviations

SOX9: [SRY (sex determining region Y)-box 9] gene; CD: Campomelic campomelic dysplasia; DSD: Disorders of sex development; ACD: Acampomelic campomelic dysplasia; PRS: Pierre Robin Sequence; TESCO: (Testis-specific enhancer of SOX9 core) region.

## Competing interest

None of the authors have any conflict of interest to disclose.

## Authors’ contributions

All authors made substantive intellectual contributions to the study and have given final approval to the manuscript. Additionally, specific author’s contributions are as follows: ACSF participated in the design of the study, performed and analyzed FISH and a-CGH data, drafted the manuscript, and finalized it. AB participated in the analysis of the molecular data, DRB, PAO and CAK acquired and interpreted clinical data. AMVM conceived the study, participated in its design and coordination, drafted the manuscript and finalized it. All authors read and approved the final manuscript.

## Pre-publication history

The pre-publication history for this paper can be accessed here:

http://www.biomedcentral.com/1471-2350/14/50/prepub

## Supplementary Material

Additional file 1: Table S1 BAC clones used as probes for chromosome 7 and 17 breakpoint mapping in Patient 1. Click here for file

Additional file 2: Table S2 BAC and PAC clones used as probes for chromosome 17 and 20 breakpoint mapping in Patient 2. Click here for file

## References

[B1] Akiyama H Chaboissier MC Martin JF Schedl A de Crombrugghe B The transcription factor Sox9 has essential roles in successive steps of the chondrocyte differentiation pathway and is required for expression of Sox5 and Sox6 Genes Dev 2002 16 2813 2828 10.1101/gad.1017802 12414734 PMC187468

[B2] Pritchett J Athwal V Roberts N Hanley NA Hanley KP Understanding the role of SOX9 in acquired diseases: lessons from development Trends Mol Med 2011 17 166 174 10.1016/j.molmed.2010.12.001 21237710

[B3] Foster JW Dominguez-Steglich MA Guioli S Kwok C Weller PA Stevanovic M Weissenbach J Mansour S Young ID Goodfellow PN Brook JD Schafer AJ Campomelic dysplasia and autosomal sex reversal caused by mutations in an SRY-related gene Nature 1994 372 525 530 10.1038/372525a0 7990924

[B4] Mansour S Hall CM Pembrey ME Young ID A clinical and genetic study of campomelic dysplasia J Med Genet 1995 32 415 420 10.1136/jmg.32.6.415 7666392 PMC1050480

[B5] Moog U Jansen NJ Scherer G Schrander Stumpel CT Acampomelic campomelic syndrome Am J Med Genet 2001 104 239 245 10.1002/ajmg.10033 11754051

[B6] Holder-Espinasse M Abadie V Cormier-Daire V Beyler C Manach Y Munnich A Lyonnet S Couly G Amiel J Pierre Robin sequence: a series of 117 consecutive cases J Pediatr 2001 139 588 590 10.1067/mpd.2001.117784 11598609

[B7] Pfeifer D Kist R Dewar K Devon K Lander ES Birren B Korniszewski L Back E Scherer G Campomelic dysplasia translocation breakpoints are scattered over 1 Mb proximal to SOX9: evidence for an extended control region Am J Hum Genet 1999 65 111 124 10.1086/302455 10364523 PMC1378081

[B8] Pop R Conz C Lindenberg KS Blesson S Schmalenberger B Briault S Pfeifer D Scherer G Screening of the 1 Mb SOX9 5′ control region by array CGH identifies a large deletion in a case of campomelic dysplasia with XY sex reversal J Med Genet 2004 41 e47 10.1136/jmg.2003.013185 15060123 PMC1735745

[B9] Leipoldt M Erdel M Bien-Willner GA Smyk M Theurl M Yatsenko SA Lupski JR Lane AH Shanske AL Stankiewicz P Scherer G Two novel translocation breakpoints upstream of SOX9 define borders of the proximal and distal breakpoint cluster region in campomelic dysplasia Clin Genet 2007 71 67 75 17204049 10.1111/j.1399-0004.2007.00736.x

[B10] Benko S Fantes JA Amiel J Kleinjan DJ Thomas S Ramsay J Jamshidi N Essafi A Heaney S Gordon CT McBride D Golzio C Fisher M Perry P Abadie V Ayuso C Holder-Espinasse M Kilpatrick N Lees MM Picard A Temple IK Thomas P Vazquez MP Vekemans M RoestCrollius H Hastie ND Munnich A Etchevers HC Pelet A Farlie PG Fitzpatrick DR Lyonnet S Highly conserved non-coding elements on either side of SOX9 associated with Pierre Robin sequence Nat Genet 2009 41 359 364 10.1038/ng.329 19234473

[B11] Lecointre C Pichon O Hamel A Heloury Y Michel-Calemard L Morel Y David A Le Caignec C Familial acampomelic form of campomelic dysplasia caused by a 960 kb deletion upstream of SOX9 Am J Med Genet 2009 149A 1183 1189 10.1002/ajmg.a.32830 19449405

[B12] Jakubiczka S Schröder C Ullmann R Volleth M Ledig S Gilberg E Kroisel P Wieacker P Translocation and deletion around SOX9 in a patient with acampomelic campomelic dysplasia and sex reversal Sex Dev 2010 4 143 149 10.1159/000302403 20453475

[B13] Benko S Gordon CT Mallet D Sreenivasan R Thauvin-Robinet C Brendehaug A Thomas S Bruland O David M Nicolino M Labalme A Sanlaville D Callier P Malan V Huet F Molven A Dijoud F Munnich A Faivre L Amiel J Harley V Houge G Morel Y Lyonnet S Disruption of a long distance regulatory region upstream of SOX9 in isolated disorders of sex development J Med Genet 2011 48 825 830 10.1136/jmedgenet-2011-100255 22051515

[B14] White S Ohnesorg T Notini A Roeszler K Hewitt J Daggag H Smith C Turbitt E Gustin S van den Bergen J Miles D Western P Arboleda V Schumacher V Gordon L Bell K Bengtsson H Speed T Hutson J Warne G Harley V Koopman P Vilain E Sinclair A Copy number variation in patients with disorders of sex development due to 46,XY gonadal dysgenesis PLoS One 2011 6 e17793 10.1371/journal.pone.0017793 21408189 PMC3049794

[B15] Cox JJ Willatt L Homfray T Woods CG A SOX9 duplication and familial 46, XX developmental testicular disorder N Engl J Med 2011 364 91 93 10.1056/NEJMc1010311 21208124

[B16] Vetro A Ciccone R Giorda R Patricelli MG Della Mina E Forlino A Zuffardi O XX males SRY negative: a confirmed cause of infertility J Med Genet 2011 48 710 712 10.1136/jmedgenet-2011-100036 21653197 PMC3178810

[B17] Refai O Friedman A Terry L Jewett T Pearlman A Perle MA Ostrer H De novo 12;17 translocation upstream of SOX9 resulting in 46, XX testicular disorder of sex development Am J Med Genet 2010 152A 422 426 10.1002/ajmg.a.33201 20082466

[B18] Kurth I Klopocki E Stricker S van Oosterwijk J Vanek S Altmann J Santos HG van Harssel JJ de Ravel T Wilkie AO Gal A Mundlos S Duplications of noncoding elements 5′ of SOX9 are associated with brachydactyly-anonychia Nat Genet 2009 2009 41 862 863 19639023 10.1038/ng0809-862

[B19] Gordon CT Tan TY Benko S Fitzpatrick D Lyonnet S Farlie PG Long-range regulation at the SOX9 locus in development and disease J Med Genet 2009 46 649 656 10.1136/jmg.2009.068361 19473998

[B20] Velagaleti GV Bien-Willner GA Northup JK Lockhart LH Hawkins JC Jalal SM Withers M Lupski JR Stankiewicz P Position effects due to chromosome breakpoints that map approximately 900 Kb upstream and approximately 1.3 Mb downstream of SOX9 in two individuals with campomelic dysplasia Am J Hum Genet 2005 76 652 662 10.1086/429252 15726498 PMC1199302

[B21] Bagheri-Fam S Barrionuevo F Dohrmann U Gunther T Schule R Kemler R Mallo M Kanzler B Scherer G Long-range upstream and downstream enhancers control distinct subsets of the complex spatiotemporal Sox9 expression pattern Dev Biol 2006 291 382 397 10.1016/j.ydbio.2005.11.013 16458883

[B22] Sekido R Lovell-Badge R Sex determination involves synergistic action of SRY and SF1 on a specific Sox9 enhancer Nature 2008 453 930 934 10.1038/nature06944 18454134

[B23] Tommerup N Schempp W Meinecke P Pedersen S Bolund L Brandt C Goodpasture C Guldberg P Held KR Reinwein H Saugstad OD Scherer G Skjeldal O Toder R Westvik J van der Hagen CB Wolf U Assignment of an autosomal sex reversal locus (SRA1) and campomelic dysplasia (CMPD1) to 17q24.3-q25.1 Nat Genet 1993 4 170 174 10.1038/ng0693-170 8348155

[B24] Wagner T Wirth J Meyer J Zabel B Held M Zimmer J Pasantes J Bricarelli FD Keutel J Hustert E Wolf U Tommerup N Schempp W Scherer G Autosomal sex reversal and campomelic dysplasia are caused by mutations in and around the SRY-related gene SOX9 Cell 1994 79 1111 1120 10.1016/0092-8674(94)90041-8 8001137

[B25] Ninomiya S Narahara K Tsuji K Yokoyama Y Ito S Seino Y Acampomelic campomelic syndrome and sex reversal associated with de novo t(12;17) translocation Am J Med Genet 1995 56 31 34 10.1002/ajmg.1320560109 7747782

[B26] Ninomiya S Isomura M Narahara K Seino Y Nakamura Y Isolation of a testis-specific cDNA on chromosome 17q from a region adjacent to the breakpoint of t(12;17) observed in a patient with acampomeliccampomelic dysplasia and sex reversal Hum Mol Genet 1996 5 69 72 10.1093/hmg/5.1.69 8789441

[B27] Young ID Zuccollo JM Maltby EL Broderick NJ Campomelic dysplasia associated with a de novo 2q;17q reciprocal translocation J Med Genet 1992 29 251 252 10.1136/jmg.29.4.251 1583645 PMC1015925

[B28] Wunderle VM Critcher R Hastie N Goodfellow PN Schedl A Deletion of longrange regulatory elements upstream of SOX9 causes campomelic dysplasia Proc Natl Acad Sci USA 1998 95 10649 10654 10.1073/pnas.95.18.10649 9724758 PMC27949

[B29] Wirth J Wagner T Meyer J Pfeiffer RA Tietze HU Schempp W Scherer G Translocation breakpoints in three patients with campomelic dysplasia and autosomal sex reversal map more than 130 kb from SOX9 Hum Genet 1996 97 186 193 10.1007/BF02265263 8566951

[B30] Sobreira NL Gnanakkan V Walsh M Marosy B Wohler E Thomas G Hoover-Fong JE Hamosh A Wheelan SJ Valle D Characterization of complex chromosomal rearrangements by targeted capture and next-generation sequencing Genome Res 2011 21 1720 1727 10.1101/gr.122986.111 21890680 PMC3202288

[B31] Hill-Harfe KL Kaplan L Stalker HJ Zori RT Pop R Scherer G Wallace MR Fine mapping of chromosome 17 translocation breakpoints > or = 900 kb upstream of SOX9 in acampomelic campomelic dysplasia and a mild, familial skeletal dysplasia Am J Hum Genet 2005 76 663 671 10.1086/429254 15717285 PMC1199303

[B32] Fukami M Tsuchiya T Takada S Kanbara A Asahara H Igarashi A Kamiyama Y Nishimura G Ogata T Complex genomic rearrangement in the SOX9 5′ region in a patient with Pierre Robin sequence and hypoplastic left scapula Am J Med Genet 2012 158A 1529 1534 10.1002/ajmg.a.35308 22529047

[B33] Huang B Wang S Ning Y Lamb AN Bartley J Autosomal XX sex reversal caused by duplication of SOX9 Am J Med Genet 1999 87 349 353 10.1002/(SICI)1096-8628(19991203)87:4<349::AID-AJMG13>3.0.CO;2-N 10588843

